# Analysis of Orthogonal Coupling Structure Based on Double Three-Contact Vertical Hall Device

**DOI:** 10.3390/mi10090610

**Published:** 2019-09-14

**Authors:** Rongshan Wei, Yuxuan Du

**Affiliations:** College of Physics and Information Engineering, Fuzhou University, Fuzhou 350116, Fujian, China; n161120017@fzu.edu.cn

**Keywords:** orthogonal coupling, offset voltage, vertical Hall device, conformal mapping

## Abstract

A vertical Hall device is an important component of 3D Hall sensors, used for detecting magnetic fields parallel to the sensor surface. The Hall devices described in existing research still have problems, such as large offset voltage and low sensitivity. Aiming to solve these problems, this study proposes a double three-contact vertical Hall device with low offset voltage, and a conformal mapping analysis method to improve the sensitivity of the device. Secondly, an orthogonal coupling structure composed of two sets of double three-contact vertical Hall devices is proposed, which further reduces the offset voltage of the device. Finally, the TCAD simulation software was used to analyze the performance of the devices, and an existing vertical Hall device was compared to ours. The results show that the orthogonal coupling structure in this study exhibits better performance, reaching an average voltage sensitivity of 17.5222 mV/VT and an average offset voltage of about 0.075 mV. In addition, the structure has the same magnitude of offset voltage in the four phases of the rotating current method. This characteristic enables the back-end circuit to more accurately filter out the offset voltage and acquire the Hall signal.

## 1. Introduction

The Hall device is the most critical part of a 3D Hall sensor for magnetoelectric conversion [1,2]. The vertical Hall device is used to detect the magnetic field parallel to the sensor surface, and is still in the development stage due to its special structure. Structurally, the Hall device includes a set of bias electrodes and a set of Hall electrodes [3,4]. In cases where a bias voltage and a magnetic field are applied, the Hall signal is output by the two Hall electrodes.

In recent years, in order to improve the performance of the device, five-contact, four-contact and six-contact structures have gradually appeared [5,6,7,8,9], but they still have problems of high offset voltage and low sensitivity. Aiming to solve these problems, this paper proposes a double three-contact vertical Hall device structure and its conformal mapping analysis method to improve the performance of the device. Furthermore, considering the effects of temperature and strain [10,11,12], this paper proposes an orthogonal coupling structure based on double three-contact vertical Hall devices to further reduce the offset voltage. Finally, several structures studied in this paper were constructed using TCAD software (version 12.0.0.58849, Silvaco International, Santa Clara, CA, USA), and compared with the research results in recent years to verify whether the orthogonal coupling structure exhibits better performance.

## 2. Methods

### 2.1. Double Three-Contact Vertical Hall Device and Orthogonal Coupling Structure

**Figure 1:**
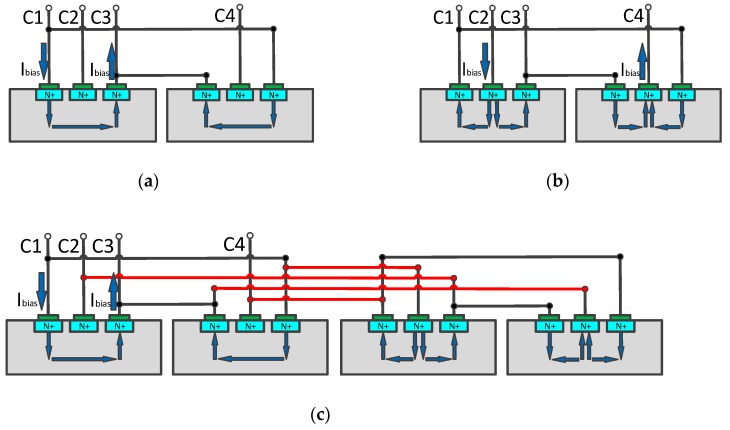
The current flow path: (**a**) the double three-contact vertical Hall device in the phase 0; (**b**) the double three-contact vertical Hall device in the phase 90; (**c**) the orthogonal coupling structure in the phase 0.

The structure of the double three-contact vertical Hall device is shown in Figure 1a. The two three-contact structures were mirror images of each other, and the overall structure of the device was completely symmetrical, which can help reduce the initial offset [6]. When the rotating-current [13,14] method was applied, the current flow in the phase 0 and 90 was indicated by the arrows in Figure 1a,b respectively. The current flow path was symmetrical, which was advantageous for reducing the offset voltage in different phases. However, the current flow path of the device between different phases will cause a difference in the amplitude of the offset voltage.

In order to solve this problem, this paper proposes an orthogonal coupling structure composed of two sets of the double three- contact devices, as shown in Figure 1c. In either phase of the structure, the two sets of double three-contact devices are in a state of 90 phase difference. This feature is beneficial to further suppress the magnitude of the offset voltage [15,16,17] and balance the amplitude of the offset voltage between the different phases, so that the back-end signal processing circuit can more accurately filter out the offset voltage and extract the Hall signal.

### 2.2. Conformal Mapping Principle

**Figure 2:**
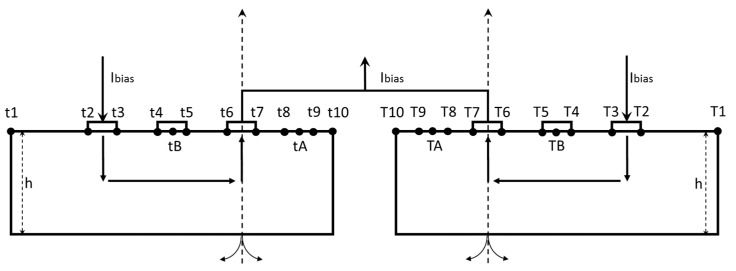
Double three-contact structure.

Using the conformal mapping method, the parameters affecting the performance of the double three-contact device were analyzed, and the performance optimization of the orthogonal coupling structure was realized. However, existing conformal mapping was mainly applied to five- or four-contact device [18]. The double three-contact device structure proposed includes two independent N wells, so the structure cannot be directly deployed, and needs to be analyzed separately. The coordinate system of the structure was established as shown in Figure 2.

On the other hand, the conventional five- or four-contact structure includes a set of bias electrodes and a set of Hall electrodes to make the conformal mapping process smooth. In this study, since each single three-contact structure needed to be separately analyzed, a necessary Hall electrode was missing, so the missing electrode needed to be virtualized.

In the analysis of a vertical Hall device, the N well with a finite depth and width needs to be transformed to the lower half of the W plane, and the electrodes are distributed on the real axis of the plane. In the structure shown in Figure 2, coordinate systems were established at the midpoints of the two output electrodes of the three-contact structure, and the boundary points of the respective electrodes and N well were sampled. The Jacobian elliptic integration was used, as in Equation (1), to complete the transformation step:

(1) $$w=\mathrm{sn}[\frac{t}{A},k^{2}]$$

This is the inverse function of the first type of elliptic integration, and is a two-period meromorphic function, where k is the elliptical mode. The values of A and k can be determined by the following equations:

(2) $$A=\frac{\left|t_{1}-t_{10}\right|}{2F\langle a\sin(1)|k^{2}\rangle }$$

(3) |t1−t10|2h=−F〈asin(1)|k2〉Im〈F[asin(1k)|k2]〉

In addition, Equation (5) is needed to normalize the sampling points [19]:

(4) $$W=\frac{2w}{\left|w_{7}-w_{6}\right|}$$

**Figure 3:**
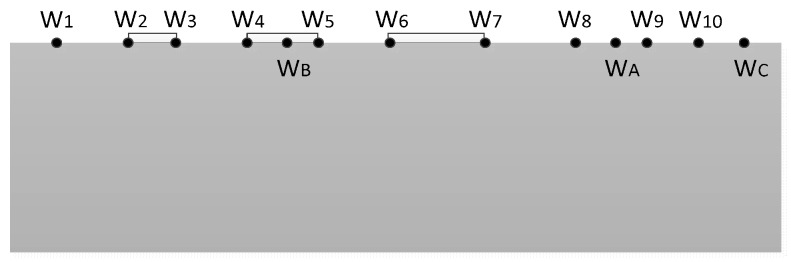
Transformation result in W plane.

Taking the three-contact structure on the left-hand panel of Figure 2 as an example, the transformation result is shown in Figure 3. The N well was expanded to the lower half of the W plane along the direction from the origin to the sampling point of t_15_. The original t_15_ sample point was transformed to W_15_, which was located at infinity on the W plane. t_8_ and t_9_ were the sampling points of the virtual electrode; that is, it was assumed that there is one electrode between t_8_ and t_9_. t_A_ was the sampling point between t_8_ and t_9_ and t_B_ is the sampling point between t_4_ and t_5_. After the transformation was completed, W_A_ was still between W_8_ and W_9_ and W_B_ was between W_4_ and W_5_.

Finally, the Schwarz-Christoffel transform maps the W half-plane and each sample point to the Z plane:

(5) $$Z=H\int_{0}^{W}\frac{\left(u-W_{A})(u-W_{B}\right)}{f_{1}f_{2}f_{3}}du$$

where

(6) $$f_{1}={\left(u-W_{7}\right)}^{\alpha^{+}}{\left(u-W_{8}\right)}^{\alpha^{-}}{\left(u-W_{9}\right)}^{\alpha^{+}}$$

(7) $$f_{2}={\left(u-W_{6}\right)}^{\alpha^{-}}{\left(u-W_{5}\right)}^{\alpha^{+}}{\left(u-W_{4}\right)}^{\alpha^{-}}$$

(8) $$f_{3}={\left(u-W_{2}\right)}^{\alpha^{-}}{\left(u-W_{3}\right)}^{\alpha^{+}}$$

(9) $$\alpha^{+}=\frac{1}{2}+\frac{\beta }{\pi }$$

(10) $$\alpha^{-}=\frac{1}{2}-\frac{\beta }{\pi }$$

*β* is the Hall angle and satisfies

(11) $$\tan(\beta )=\mu_{h}B$$

where *μ_h_* is the carrier mobility [20], the polarity of which is determined by the nature of the carrier. B is the magnetic induction in which the device is located. After the transformation process, each sampling point was mapped to the Z plane and connected to form a quadrilateral. By adjusting the values of W_A_ and W_B_, the result of the transformation can be changed from quadrilateral to parallelogram, and the two sides of the parallelograms that are parallel to the real axis are equal in size. H is the correction factor, and its value can be determined by the following equation:

(12) $$H=-\frac{W_{2}e^{-i(\frac{\pi }{2}-\beta )}}{W_{c}}$$

where W_C_ is located on the real axis of the W plane. 

The sides in which Z_2_ and Z_3_ are located corresponded to the bias input electrode of the original structure. In the Z plane the bias current also flowed from the side. The sides in which Z_6_ and Z_7_ are located corresponded to the bias output electrodes. The bias current flowed out from the side and the current direction was parallel to the hypotenuse side. The Hall electrode and virtual electrode in the original three-contact structure corresponded to the points on the two hypotenuse sides of the parallelogram, respectively. The difference in height between the two sets of points reflected the properties of the Hall device, including the following:

Geometric factor:

(13) $$G=\frac{1}{\left|\sin(\beta )\right|}\frac{\left||Z_{8}-Z_{7}|-|Z_{5}-Z_{6}|\right|}{\left|Z_{7}-Z_{6}\right|}$$

Series resistance:

(14) $$R_{\mathrm{in}}=\frac{\rho }{t}\frac{1}{\left|\cos(\beta )\right|}\frac{\left|Z_{6}-Z_{3}\right|}{\left|Z_{7}-Z_{6}\right|}$$

Voltage sensitivity S_V_:

(15) $$S_{V}=\frac{G}{t}\frac{r_{h}}{qN_{D}R_{in}}.$$

where *ρ* is the resistivity of the device, which is affected by temperature, material, and doping concentration; q is the charge amount and N_D_ is the carrier concentration; t is the thickness of the Hall device.

**Figure 4:**
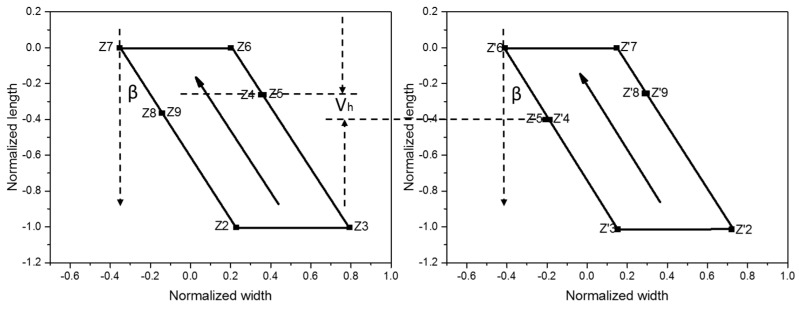
Results of conformal mapping of double three-contact vertical Hall devices.

The conformal mapping described can only be applied to the device structure of a single N well, and this study focused on the double three-contact structure. The other three-contact structure needed to be transformed using conformal mapping. The position and height difference between the two Hall electrodes in the Z plane reflected the geometric factor and device sensitivity of the double three-contact structure. The transformation results are shown in Figure 4.

By adjusting the values of the variables, such as W_A_, W_B_, and W_C_, the two parallelograms were normalized to the same size and position. By analyzing the corresponding points of the two Hall electrodes in the Z plane and their height difference, the overall property of the double three-contact device structure can be calculated. The geometric factor G_eff_ can also be calculated as follows:

(16) $$G_{eff}=\frac{1}{\left|\sin(\beta )\right|}\frac{\left||Z_{5}^{\prime }-Z_{6}^{\prime }|-|Z_{5}-Z_{6}|\right|}{\left|Z_{7}-Z_{6}\right|}.$$

### 2.3. Structural Analysis and Comparison

**Figure 5:**
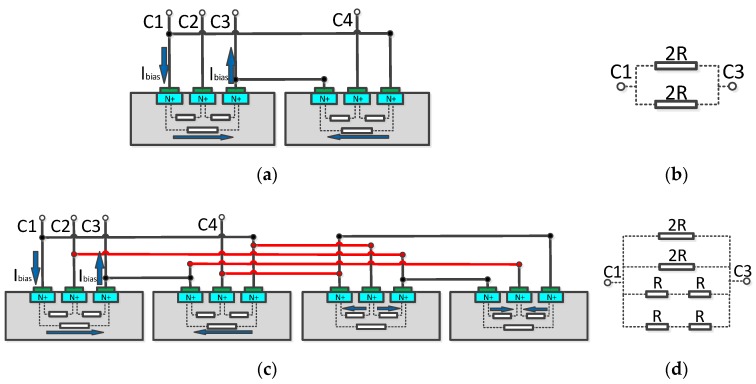

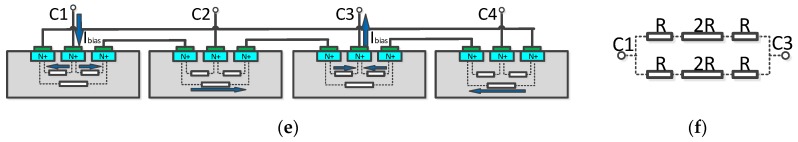
(**a**) The current flow path for a double three-contact device at phase 0; (**b**) the equivalent model for a double three-contact device; (**c**) the current flow path for the orthogonal coupling structure at phase 0; (**d**) the equivalent model for the orthogonal coupling structure; (**e**) the current flow path for a low-offset structure at phase 0; (**f**) the equivalent model for a low-offset structure.

This section analyzes and compares the equivalent models [17,21] of several structures based on three-contact devices. Assuming that in the ideal case the distances between adjacent electrodes are the same, the equivalent resistance between adjacent electrodes is R, as shown by the short white square in Figure 5. The equivalent resistance between the two outer electrodes of the three-contact device is 2R, as shown by the long white square in Figure 5.

Figure 5a shows the current flow path for a double three-contact device at phase 0, and Figure 5b shows the equivalent model for this case. When a constant voltage *V*_bias_ is applied between ports C1 and C3, the total current *I*_bias1_ flowing through the device is:

(17) $$I_{\mathrm{bias}1}=\frac{V_{\mathrm{bias}}}{2R//2R}=\frac{V_{\mathrm{bias}}}{R}$$

In the equivalent model, each branch corresponds to a branch current flow path, so the current in a single branch is (1/2) *I*_bias1_.

Figure 5c shows the current flow path for the orthogonal coupling structure at 0 phase, and Figure 5d is the equivalent model in this case. When a constant voltage *V*_bias_ is applied between ports C1 and C3, the total current *I*_bias2_ is:

(18) $$I_{\mathrm{bias}2}=\frac{V_{\mathrm{bias}}}{2R//2R//(R+R)//(R+R)}=2\frac{V_{bias}}{R}$$

The equivalent model of the orthogonal coupling structure consists of four branches, and the equivalent impedance of the branches are the same, so the branch current is (1/4) *I*_bias2_. It can be seen that in the ideal case, the branch currents of the double three-contact structure and the orthogonal coupling structure are the same, so the two structures will have similar output voltages.

In contrast, Figure 5e shows a low-offset structure [22,23,24] and its current flow path at 0 phase. In this structure, the two electrodes closest to each other between adjacent three-contact structures are connected by wires, and the two electrodes of the outermost side of the overall structure are connected. Therefore, when a constant voltage *V*_bias_ is applied between ports C1 and C3, current needs to flow through the three devices to reach port C3. The equivalent model is shown in Figure 5f. The total current *I*_bias3_ is:

(19) $$I_{\mathrm{bias}3}=\frac{V_{\mathrm{bias}}}{\left(R+2R+R)//(R+2R+R\right)}=\frac{V_{\mathrm{bias}}}{2R}$$

The low offset structure has two branches, so its branch current is (1/2) *I*_bias3_. It can be seen that, under ideal conditions, the branch current of the orthogonal coupling structure is twice that of the low offset structure, and the impedance of the branch is lower, so the orthogonal coupling structure has higher output voltage and device sensitivity.

## 3. Results and Discussion

### 3.1. Analysis of Conformal Mapping

**Figure 6:**
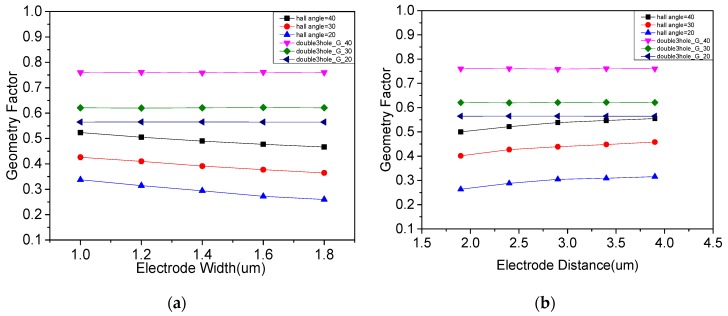
The analysis results: (**a**) effect of width of virtual electrode on device performance; (**b**) effect of position of virtual electrode on device performance.

Figure 6a shows the effect on the device performance with the width of the virtual electrode as a single variable in the case of three Hall angles. According to the data, the width variation of the virtual electrode only affected the position of the electrode mapped to the hypotenuse of the parallelogram without affecting the position of the Hall electrode mapped to the hypotenuse. Thus, for a single three-contact structure, a larger virtual electrode width will reduce its geometry factor, while the overall performance of the double three-contact device does not change. Similarly, Figure 6b shows the variation of device performance in the case of three kinds of Hall angles, taking the distance from the virtual electrode to the bias electrode as a single variable. It can be seen that with a longer distance, the geometry factor of a single three-contact structure rose slightly, but the overall geometry factor of the double three-contact device did not change. In summary, it can be considered that the concept of the virtual electrode does not affect the correctness of the conformal mapping process of the device.

**Figure 7:**
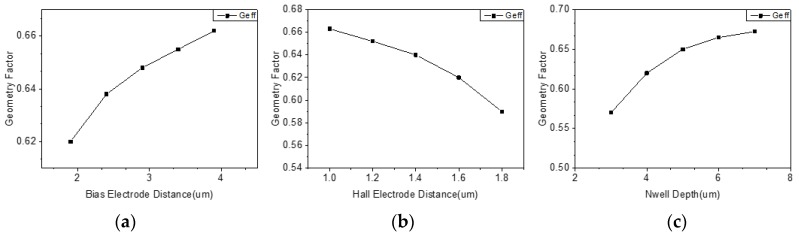
(**a**) Effect of position of bias electrode on device performance; (**b**) effect of position of Hall electrode on device performance; (**c**) effect of N-well depth on device performance.

Using the conformal mapping method of the double three-contact device, several structural parameters that may affect the performance of the device were studied. Figure 7a shows the effect of changing the distance from the bias input electrode to the Hall electrode on the performance of overall structure. It can be seen that the change of the distance affected the position at which the Hall electrode was mapped to the Z plane, and the geometry factor of the device increased as the distance increased, thereby obtaining higher sensitivity. This phenomenon is further verified in Figure 7b, which shows the effect of changing the distance from the Hall electrode to the biased output electrode on the performance of overall structure. It can be seen that the geometry factor of the device decreased as the distance increased. Figure 7c shows the effect of N-well depth on device performance. A larger N-well depth facilitated bias current flowing deep into the device, thus increasing the sensitivity of the device.

### 3.2. Simulation and Experimental Results

**Figure 8:**
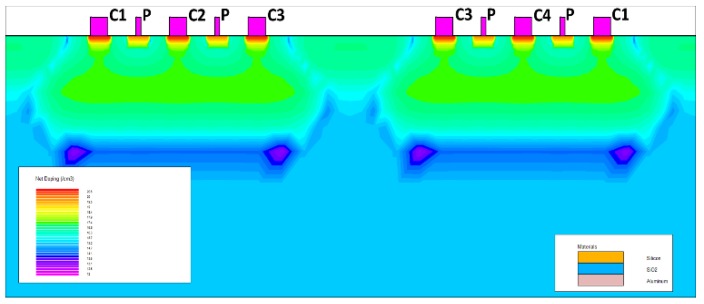
The double three-contact structure.

The double three-contact structure shown in Figure 8 is a compromise based on the conformal mapping conclusion. The distance between the contact electrodes is the same, and the distance between the Hall electrode and the two bias electrodes is shortened in order to improve sensitivity. In the figures, the same reference numerals indicate that the corresponding electrodes are connected by wires.

**Figure 9:**
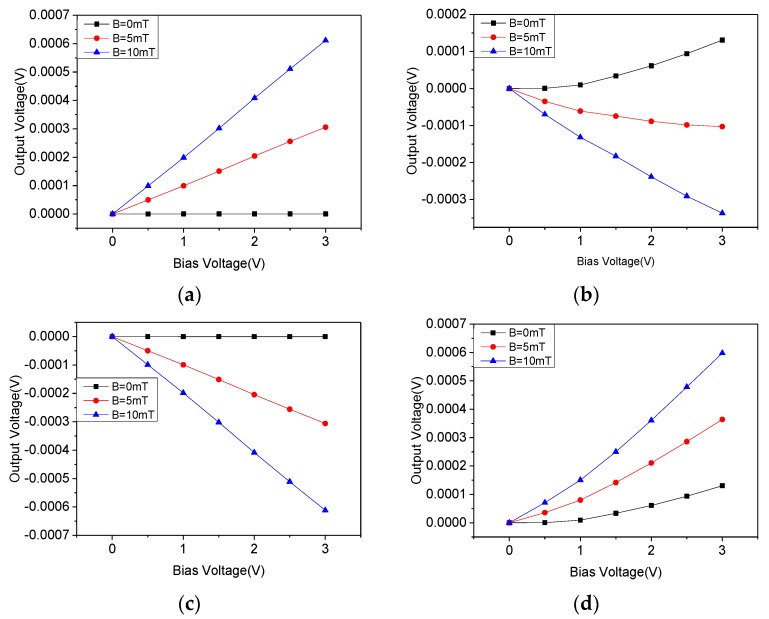
The output voltages between the Hall electrodes of the double three-contact structure. (**a**) Phase 0; (**b**) Phase 90; (**c**) Phase 180; (**d**) Phase 270.

In a magnetic field of 0 mT, 5 mT, and 10 mT, the output voltage of the double three-contact structure in four phases is as shown in Figure 9, and is represented by black, red, and blue lines, respectively.

It can be seen that the offset voltage of the double three-contact structure was still at an extremely low level in phase 0 and 180. In phase 90 and 270, the offset voltage increased with the bias voltage, but its maximum value was suppressed to about 0.13 mV.

When the bias voltage was 3 V and in a 10 mT magnetic field, the offset voltage and the voltage sensitivity of the device at different phases is shown in Table 1. The average voltage sensitivity was approximately 17.9799 mV/VT. However, this structure still had the problem that the offset voltage and sensitivity were inconsistent in four phases, which is not conducive to the extraction of the Hall signal by the back-end circuit.

**Figure 10:**
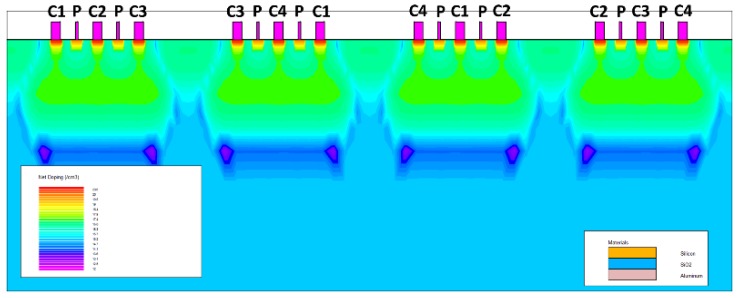
The orthogonal coupling structure.

In order to further optimize the device performance, an orthogonal coupling structure is proposed without changing the size parameters of the three-contact device. This design not only helps to further suppress the offset voltage, but also equalizes the sensitivity of the device in four phases, thereby reducing the complexity of the back-end Hall signal processing circuit. The simulation model of the orthogonal coupling structure is shown in Figure 10.

**Figure 11:**
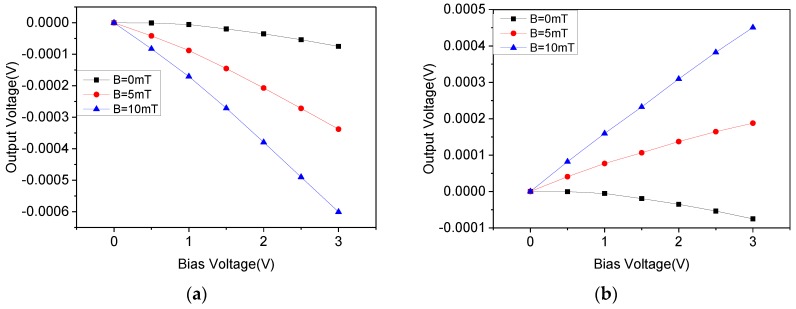

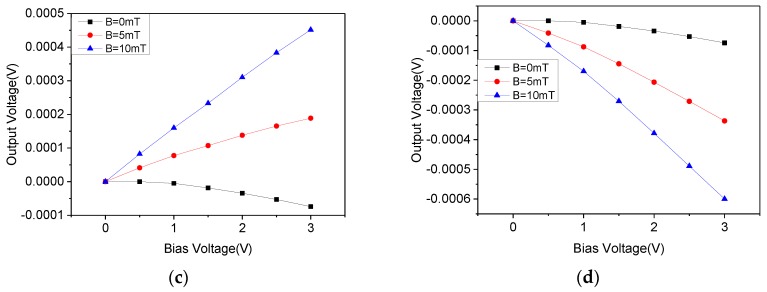
The output voltages between the Hall electrodes of the orthogonal coupled structure. (**a**) Phase 0; (**b**) Phase 90; (**c**) Phase 180; (**d**) Phase 270.

In a magnetic field of 0 mT, 5 mT, and 10 mT, the output voltage of the orthogonal coupling structure in four phases is as shown in Figure 11, which are represented by black, red, and blue lines, respectively.

It can be seen that under four phases, the offset voltage of the orthogonal coupling structure has the same trend, and the maximum value is about 0.075 mV. The offset voltage of the structure is further reduced compared to the double three-contact structure. In addition, the offset voltage values are the same in different phases, which is beneficial to improve the accuracy of the back-end circuit for filtering the offset signal and extracting the Hall signal.

When the bias voltage was 3 V and in a 10 mT magnetic field, the offset voltage and the voltage sensitivity of the device at different phases are shown in Table 2. The average voltage sensitivity was approximately 17.5222 mV/VT. It can be seen that the sensitivity of the orthogonal coupling structure did not decrease, and the sensitivity at four phases was almost the same. This feature led the signal to be processed by the rotating current circuit closer to the standard square wave signal, which is beneficial in the accurate extraction and amplification of the Hall signal by the back-end circuit.

**Figure 12:**
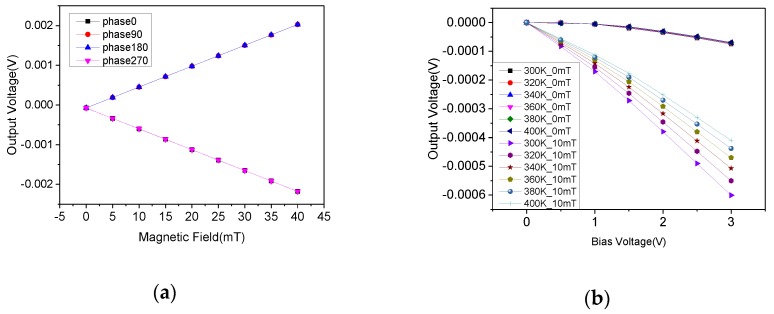
(**a**) Effect of magnetic induction on device; (**b**) Effect of temperature on device.

Figure 12a shows the relationship between the output voltage of the orthogonal coupled structure and the magnetic induction at 3 V bias voltage. It can be seen that under four phases, there is a linear trend, which makes the output voltage of the device more accurately reflect the change of the magnetic field.

Figure 12b shows the relationship between the output voltage of the device and the temperature of the orthogonal coupling structure under phase 0. It can be seen that there was no current in the device without bias voltage, so the offset voltage was hardly affected by temperature. After the bias voltage was applied, a current was generated inside the device, and the increase in temperature reduced the mobility of the carrier and Hall factor r_h_ [20]. According to Equation (15), the output voltage and sensitivity of the Hall device would also decrease, which was an overall trend regardless of the structure of the device. Table 3 shows the performance parameters of several structures involved in the previous section, and compares them with the vertical Hall device structure which appeared in recent years.

Comparing to the double three-contact structure, it can be seen that the orthogonal coupling structure greatly suppressed the offset voltage with little sensitivity reduction, and achieved the same magnitude of offset voltage and sensitivity in four phases. This feature helped improve the accuracy of the back-end circuit to extract and process Hall signals. In contrast to the traditional structure [7,17] and the low offset structure [17,22], the orthogonal coupling structure still exhibited better performance.

## 4. Conclusions

This study proposes an orthogonal coupling structure composed of two sets of double three-contact vertical Hall devices, which achieves the purpose of further reducing the offset voltage of the device while maintaining the sensitivity of the device. 

Compared with existing research, the sensitivity of the orthogonal coupling structure was still at a high level, reaching 17.5222 mV/VT, and the amplitude of the Hall signal was consistent in the four phases of the rotating current method. This characteristic makes the output signal of the rotating current circuit closer to the square wave, and the back-end circuit can extract the Hall signal more accurately. In addition, the offset voltage of the orthogonal coupling structure reached 0.075 mV, and its amplitude was consistent under the four phases of the rotating current method. This feature helps the back-end circuitry to filter out the offset voltage more thoroughly and avoid residual offsets.

The study found that the output signal of the device was affected by temperature, so in future work, calibration circuits need to be designed to correct the effect of operating temperature.

## Figures and Tables

**Table 1:** The offset voltages and voltage sensitivity of the double three-contact structure.

Performance	Phase 0	Phase 90	Phase 180	Phase 270
The output voltage (V)	0.000611491	−0.0003366	−0.00061185	0.000597739
The offset voltage (V)	0.00000032	0.000130889	−0.000000336	0.000130328
The voltage sensitivity (mV/VT)	20.3724	15.583	20.3838	15.5804

**Table 2:** The offset voltages and voltage sensitivity of the orthogonal coupled structure.

Performance	Phase 0	Phase 90	Phase 180	Phase 270
The output voltage (V)	−0.00060061	0.000450623	0.000451527	−0.00059990
The offset voltage (V)	−0.00007499	−0.00007499	−0.00007418	−0.00007418
The voltage sensitivity (mV/VT)	17.5207	17.5204	17.5236	17.5240

**Table 3:** Performance comparison between different vertical Hall devices.

Structures	V_off_-max (mV)	V_off_-ave (mV)	S_V_ (mV/VT)
Double three-contact structure	0.130889	0.065468	17.9799
Orthogonal coupling structure	0.07499	0.074585	17.5222
5CVHS [17]	5	3.25	17.1
FSVHS [17]	3	3	11.6
ULOVHS [22]	0.25	0.08	11.5
LV-VHD [7]	2.3	0.9	10.42
